# Anther‐smut fungi from more contaminated sites in Chernobyl show lower infection ability and lower viability following experimental irradiation

**DOI:** 10.1002/ece3.6376

**Published:** 2020-05-26

**Authors:** Sylvie Arnaise, Jacqui A. Shykoff, Anders P. Møller, Timothy A. Mousseau, Tatiana Giraud

**Affiliations:** ^1^ Ecologie Systematique Evolution CNRS Université Paris‐Saclay Orsay France; ^2^ Department of Biological Sciences University of South Carolina Columbia SC USA

**Keywords:** disease, fungi, infection, *Microbotryum lychnidis‐dioicae*, pathogen, radiation, radioactivity, resistance, *Silene latifolia*, tolerance

## Abstract

The long‐term contamination that followed the nuclear disaster at Chernobyl provides a case study for the effects of chronic ionizing radiation on living organisms and on their ability to tolerate or evolve resistance to such radiation. Previously, we studied the fertility and viability of early developmental stages of a castrating plant pathogen, the anther‐smut fungus *Microbotryum lychnidis‐dioicae,* isolated from field sites varying over 700‐fold in degree of radioactive contamination. Neither the budding rate of haploid spores following meiosis nor the karyotype structure varied with increasing radiation levels at sampling sites. Here, we assessed the ability of the same *M. lychnidis‐dioicae* strains to perform their whole life cycle, up to the production of symptoms in the plants, that is, the development of anthers full of fungal spores; we also assessed their viability under experimental radiation. Fungal strains from more contaminated sites had no lower spore numbers in anthers or viability, but infected host plants less well, indicating lower overall fitness due to radioactivity exposure. These findings improve our understanding of the previous field data, in which the anther‐smut disease prevalence on *Silene latifolia* plants caused by *M. lychnidis‐dioicae* was lower at more contaminated sites. Although the fungus showed relatively high resistance to experimental radiation, we found no evidence that increased resistance to radiation has evolved in populations from contaminated sites. Fungal strains from more contaminated sites even tolerated or repaired damage from a brief acute exposure to γ radiation less well than those from non‐ or less contaminated sites. Our results more generally concur with previous studies in showing that the fitness of living organisms is affected by radiation after nuclear disasters, but that they do not rapidly evolve higher tolerance.

## Graphical abstract

Strains of the anther‐smut castrating fungus from more contaminated sites in Chernobyl infected host plants less well, indicating lower fitness due to radioactivity exposure. The fungus showed relatively high resistance to experimental radiation, but we found no evidence that resistance to radiation has evolved within these populations following radiation experiments. Strains from sites with higher field radiation had in contrast lower tolerance to experimental acute radiation.

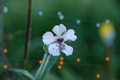

## 1 INTRODUCTION

The nuclear accident at Chernobyl in April 1986 caused widespread contamination by a large number of radionuclides that generate ionizing radiation. Such radiation is mutagenic and toxic for living organisms either by direct damage to the DNA or by generating reactive oxygen species (ROS) within cells that attack the DNA and other cellular components (Einor, Bonisoli‐Alquati, Costantini, Mousseau, & Møller, 2016). Much DNA damage due to radiation can be repaired, and genome integrity following radiation damage can be restored by cellular repair systems. Species vary greatly in their ability to repair radiation damage (review in Garnier‐Laplace et al., 2013; Gladyshev & Meselson, 2008; Lehmann, Boratynski, Mappes, Mousseau, & Møller, 2016; Rivasseau et al.., 2013) as do populations (Azizyan & Ter‐Hovhannesyan, 2010; Cordeiro, Marques, & Veiga‐Neto, 1973) or lines within species (Vaisnav et al., 2014).

Radioactive contamination in and around the Chernobyl accident site affects natural populations and communities of many different taxa, increasing mutation rates in birds and plants (Ellegren, Lindgren, Primmer, & Møller, 1997; Kovalchuk, Dubrova, Arkhipov, Hohn, & Kovalchuk, 2000; Møller, Bonisoli‐Alquati, & Mousseau, 2013; Møller & Mousseau, 2015), decreasing individual fertility (Møller, Bonisoli‐Alquati, Mousseau, & Rudolfsen, 2012) and increasing mortality (Møller, Bonisoli‐Alquati, Rudolfsen, & Mousseau, 2012) in birds, and modifying community composition (Møller & Mousseau, 2011) and influencing ecosystem functioning (Mousseau, Milinevsky, Kenney‐Hun, & Møller, 2014). Some studies have suggested that diverse organisms, including bacteria, plants, and birds, have adapted to radiation following long‐term exposure (Kovalchuk et al., 2003, Kovalchuk, Abramov, Pogribny, & Kovalchuk, 2004, Galván et al., 2014, Ruiz‐González et al., 2016; see Geras'kin, Evseeva, & Oudalova, 2013, Møller & Mousseau, 2016 for reviews). Indeed, populations from sites with high natural levels of ionizing radiation (Azizyan & Ter‐Hovhannesyan, 2010; Cordeiro et al., 1973) or from sites contaminated with radioactivity by human activity sometimes show greater resistance to radiation (Boubriak et al., 2008; Geras’kin et al., 2005; Ruiz‐González et al., 2016) or other mutagens (Kovalchuk et al., 2004) than do those from more pristine environments. These findings, together with evolution experiments (Byrne et al., 2014), indicate that radiation resistance can evolve in response to long‐term exposure. However, Ukrainian and Russian studies at Chernobyl were generally based on comparisons between one single contaminated and one control site, making it impossible to draw any conclusion (Møller & Mousseau, 2016). Therefore, there is no convincing evidence that shows an overall tendency for radiation resistance to evolve in response to human‐induced radioactive exposure (Syomov, Ptitsyna, & Sergeeva, 1992, Pimentel, Levine, Cruces, & Salceda, 2003, Geras’kin et al., 2011; see Geras'kin et al., 2013 and Møller & Mousseau, 2016 for reviews). Response may vary with the type of radiation. For example, resistance appears to evolve in response to selection imposed by β and γ, but not to α radiation (Boubriak et al., 2008; Syomov et al., 1992). Thus, the question as to whether and under which circumstances radioresistance evolves remains open, demanding further studies with well‐designed experiments. However, one may in contrast expect organisms exposed to chronic radiation to have accumulated DNA damage that renders them particularly sensitive to further damage by radiation exposure. Addressing these questions will be particularly important for predicting long‐term outcomes of contamination from human activities.

Fungi are particularly interesting in this context. Fungi with melanized hyphae show high resistance to γ radiation (Saleh et al., 1988). Several fungi have been isolated from within the Chernobyl reactor 4 (Zhdanova, Zakharchenko, Vember, & Nakonechnaya, 2000), and the reactor strains of the filamentous fungus *Alternaria alternata* show far higher resistance to γ radiation in laboratory experiments than samples from other sites (Mironenko, Alekhina, Zhdanova, & Bula, 2000). Furthermore, the corn‐smut fungus *Ustilago maydis* is the eukaryote with the greatest known resistance to ionizing radiation (Holliday, 2004). The insect‐vectored anther‐smut fungus, *Microbotryum lychnidis‐dioicae*, occurs at Chernobyl, where it castrates its host plant *Silene latifolia*. We have previously investigated patterns of infection in the field and found low prevalence at more contaminated sites that appeared in part due to a paucity of vectors in areas of intermediate contamination, and in part due to poor transmission independent of vector availability at the most contaminated sites (Aguileta et al., 2016). Genome sequence data even revealed a tendency for lower substitution rates at the more contaminated sites, suggesting more efficient purifying selection despite the smaller effective population sizes associated with lower prevalence (Aguileta et al., 2016). Though apparently lacking melanin as a protective mechanism, *M. lychnidis‐dioicae* from more contaminated sites suffered no change in viability, budding rates of haploid sporidia, mutation rates, or chromosomal aberrations, as assessed from genome sequencing and fertility estimates (Aguileta et al., 2016).

Here, we explore the ability of these same strains, isolated from a gradient of contamination levels, to infect plants and, for a subset of these strains, to resist single high doses of radiation under controlled conditions. More specifically, we pose the following questions relating to resistance to ionizing radiation: (a) Do strains isolated from areas with high levels of contamination have lower fitness, that is, lower ability to infect their host plants, due to radiation received during their lifetime? To address this question, we experimentally inoculated plants with fungal strains from sites with a 700‐fold range of radiation levels. (b) Do populations isolated from areas with higher contamination level show higher or lower tolerance to ionizing radiation? This examines whether chronic exposure over the three decades since the Chernobyl accident has allowed increased tolerance to evolve or in contrast rendered the fungus more sensitive to acute radiation. To address this question, we submitted some of our fungal strains to experimental high radiation doses from a caesium‐137 γ source. Caesium‐137 was one of the common isotopes released in the Chernobyl explosion, and it is and will remain, for the next few hundred years, one of the principal radioactive contaminants near both the Chernobyl and Fukushima accident sites. Assessment of biological data and their significance relies on three different frequentist approaches that either use significance testing (Everitt, 2002; Neyman, 1937), Akaike's information criterion (Akaike, 1981; Burnham & Anderson, 2002), or comparison of effect sizes (Cohen, 1988). These are very general approaches, and there is no consensus as to which approach is “best.” Here, we present and discuss effect sizes that have the great advantage of providing judgments of whether effects can be considered to be small (accounting for 1% of the variance), intermediate (accounting for 9% of the variance), or large (accounting for 25% of the variance; Cohen, 1988).

## 2 MATERIALS AND METHODS

### 2.1 Sampling

During regular fieldwork from 2013 to 2014 in and around the Chernobyl exclusion zone, we collected unopened buds from diseased *Silene latifolia* into individual paper or glassine envelopes and placed them in plastic (Ziplock) bags containing silica gel (Table A1). GPS coordinates were recorded for each site where diseased plants were collected, and the level of ambient radiation was measured at ground level three to five times at each location with a hand‐held dosimeter (Inspector; SE International, Summertown, TN). Mean radiation estimates from these data were then used for each location. Measurements of ambient dose are strongly positively correlated with internal dose for a wide range of organisms (Omar‐Nazir et al., 2018). For the following experiments, we used spores from 18 different strains—15 from Chernobyl, from areas with radioactive contamination varying 700‐fold, from 0.03 µSv/hr to 21.03 µSv/hr, and three control strains from uncontaminated sites but belonging to the same Central‐Eastern European genetic cluster as the Chernobyl strains (Vercken et al., 2010). Though we have some samples from highly contaminated sites, infection is rarer and prevalence is lower with increasing contamination (Aguileta et al., 2016). Therefore, the frequency distribution of samples across contamination levels is skewed, with more observations at lower contamination. When several strains were collected in some populations (same first number in their ID in Table A1), they were collected from different plants and therefore represent different individuals as the fungus has to undergo sex before infecting a new plant (Schäfer, Kemler, Bauer, & Begerow, 2010).

### 2.2 Inoculation experiment

Seeds of *Silene latifolia* were harvested from a local population growing on the Paris‐Saclay University campus (Orsay, France) in summer 2015, before French laws on Nagoya protocols were issued. Seeds were surface‐sterilized in a solution containing 12 g/L calcium hypochlorite and 4 g/L sodium hydroxyl for 20 min, then washed with the sterilizing solution diluted 10 times with sterile tap water. Batches of approximately 50 seeds were then spread onto each of 38 petri dishes (9 cm diameter) containing 1% water agar (two petri dishes for each of 18 fungal strains plus two control petri dishes without fungal inoculation; Table A1). On 8 March 2016, 36 of the petri dishes were inoculated, two with each of the 18 fungal strains as follows. To ensure that we were working with single diploid fungal genotypes from each collection site, we used spores from a single flower for each inoculation treatment, because a single fungal genotype parasitizes each stem, and hence, only one genotype is present per flower, even of plants that are infected with several fungal strains at the same time (López‐Villavicencio et al., 2007). For each of the 18 fungal strains used for inoculation, four anthers from a single unopened bud were placed in a 2‐mL Eppendorf tube with 1 ml of sterile tap water and vortexed. Then, 500 µl of this spore suspension was added to two petri dishes containing seeds and agitated gently to ensure an even distribution of the spores. Two petri dishes were left without fungal spores as controls. All petri dishes were left at room temperature (approximately 20°C) with ambient light (approximately 12‐hr daylight at that time of year), near a window, for 2 weeks (i.e., under indoor conditions of temperature variation and light). Sterile tap water was added as needed. This inoculation protocol allows seeds to germinate in a suspension of teliospores and sporidia, which are produced when teliospores germinate. Dikaryotic hyphae produced after sporidia conjugation then infect the seedlings, and plants become systemically infected. The two petri dishes used per strain did not represent statistical replicates and were only used to ensure sufficient inoculated seedlings that were then planted and randomized in the greenhouse. Each fungal isolate associated with the level of contamination measured at its collection site was represented by a single estimate of percentage of diseased plants. Though spore number per anther may vary, possibly even as a function of contamination level at the collection site, we consider that all inoculation treatments delivered a large surplus of spores with regard to the limiting infection dose, since previous counts, admittedly on plants from uncontaminated areas (Kaltz & Shykoff, 2002), found approximately 2 × 10^6^ spores per anther. Therefore, infection success was unlikely to be limited by spore number. Spore viability, and ability to germinate and undergo meiosis to produce haploid spores that can replicate, conjugate, and infect were probably more important.

Two weeks after seed inoculation, between 30 and 60 seedlings for each inoculated strain and for the control treatment were planted into 0.1‐L plastic pots containing saturated potting soil. The variation in the number of seedlings was due to the contamination of a couple of petri dishes by a damping‐off fungus. Each seedling was covered with a small plastic cap for 3 days to maintain high humidity and individually labeled with the inoculated fungal strain. After 3 days, the caps were removed and plants were kept on a greenhouse bench in an unheated greenhouse with natural light (approximately 12 hr of daylight in late March) and watered ad libitum until they had formed rosettes of about 6 leaves. Then, pots were randomized across inoculation treatments and placed directly onto the natural soil floor in the main compartment of our experimental greenhouse. Thus, it was unnecessary to transplant the plants into larger pots because their roots could pierce the plastic pots. As soon as the first plants bolted, starting on 11 May 2016, plants were observed daily except on weekends until 31 July 2016, then once per week until 1 September 2016. As each plant flowered, we noted whether it flowered healthy or diseased (Table A2) and then removed it to prevent secondary disease transmission.

### 2.3 Irradiation experiment

Haploid yeast‐like sporidia of *M. lychnidis‐dioicae* can be readily cultured on nutrient medium. Sporidial cultures for all 18 strains had previously been established from a single anther from an unopened bud to ensure that we cultivated a single diploid genotype (López‐Villavicencio et al., 2007). These teliospores were suspended in sterile tap water and plated on potato dextrose agar (PDA). Under these conditions, teliospores germinate and undergo meiosis, generating haploid meiotic products from the same diploid individual. These sporidial cultures were then frozen at −20°C in Eppendorf tubes filled with silica gel. We defrosted only nine of the 18 strains (seven strains from the Chernobyl area and two control strains; Table A3), because it was impossible to count colony numbers for survival estimates for more than nine strains the same day. We cultured the nine strains by spreading them on potato dextrose agar (PDA) plates. After 20 days at 20°C at constant light, we inoculated 10 ml of liquid medium (PDB, potato dextrose broth) with an inoculation loop and incubated PDB while agitating at 20°C for 3 days. The day before irradiation, we inoculated 0.5 ml of each culture in 10 ml of fresh PDB and incubated while being agitated at 20°C. A total of 10 μl of each of the cultures was allocated randomly across the first nine wells of each of five 96‐well microtiter flat plates. Four of these microtiter plates were allocated to radiation treatments. Samples were exposed to four different doses of radiation with a GSR_D1 (GSR Cs137/C) γ irradiator (Experimental Radiotherapy Platform, Institut Curie, Bâtiment 112, 91405 Orsay Cedex, France) equipped with a 137Cs source to deliver radiation at a dose rate of 17.178 Gy/min: 30‐min exposure, 0.515 kGy; 1‐hr exposure, 1.031 kGy; 2‐hr exposure, 2.062 kGy; and 3‐hr exposure, 3.093 kGy. The control plate (no radiation) was not exposed but was placed close to the irradiator in the same room for the same period of time as the radiation treatment plates to ensure similar environmental conditions. After treatment, 10 μl from each individual culture was serially diluted into wells containing 90 μl of PDB in a new microtiter plate to obtain dilution from 1/10 to 1/10,000. For each sample and dilution level, 5 μl of the culture was plated on each of three PDA plates. These three plates were not independent replicates and were plated to have at least one without contamination by bacteria. After 24‐hr growth at 20°C, the plates were examined and the dilution level that allowed individual colonies to be easily distinguished was chosen for each strain and treatment. Colony‐forming units (CFUs) were counted under a binocular microscope for as many of the three plates per strain and treatment as possible, some being uncountable because of contamination with bacteria. The number of viable *M. lychnidis‐dioicae* cells per initial solution was estimated by multiplying the number of CFUs by the corresponding dilution factor. Counts of the control plates, without experimental irradiation, were used as a baseline for the treatment counts, to provide estimates for the number of viable spores in each solution in the absence of effects of radiation treatments. Counts of the different plates for the same strain and treatment combinations were highly repeatable (see Results), so having fewer plates per strain should have had little effect on the accuracy of viability estimates, and did not affect sample size, because we used the mean of the different plates for each strain and treatment combination.

### 2.4 Statistical analyses

We ran statistical analyses using JMP v. 12.0 (SAS Institute). Data were transformed to obtain approximately normally distributed variables, following standard transformations (Sokal & Rohlf, 1995): arcsine transformation for proportion data, that is, the infection rates, and log transformations for cell counts and radiation at the field site where each fungal strain had been collected. Arcsine transformation is calculated by computing the arcsine of the square root of the proportion. For radiation at field site, we used the log10 of (radiation + 0.001), which greatly improved the skew, giving the least clumping of data for low values of radiation. For the viability data from the irradiation experiment, we log‐transformed the raw count data corrected by dilution for each plate. To estimate sporidia viability, we calculated the difference in the log‐transformed viable cell number between each irradiated plate and each control plate of the same strain, then calculated the mean and standard deviation of those differences. This gave a measure of the variation in this estimate.

We performed a linear regression, weighted by sample size, of the arcsine‐transformed infection rates of our greenhouse‐inoculated plants on the log radiation at the field site where each fungal strain had been collected to test whether infection success under experimental conditions varied with level of radiation exposure where the fungal strain originated.

We performed one‐way ANOVAs for testing the repeatability of cell counts for the repeat plates within treatment and strain combinations to check that our counting method was reliable.

To test whether radiation level at the field site where the fungal strains had been collected impacted the inherent viability or replication rates of their haploid sporidia, we regressed the mean of log counts of sporidia in the control treatment without experimental radiation on the log of the field radiation level. We performed ANCOVA to test the effect of irradiation treatment on sporidia viability, with radiation levels at the field sites of collection of the strains as a covariate. This permitted us to ask the questions whether the viability of haploid sporidia differed depending on the experimental irradiation dose, whether fungal strains collected at sites differing in ambient radiation dose rate varied in the viability of their haploid sporidia, and whether there was an interaction between the factor and the covariate.

We further calculated and reported effect sizes for all statistical analyses converting F‐statistics to Pearson's product–moment correlation coefficients. This was done with Pearson *r* = (*F*/(*F* + *df* denominator) (Rosenthal, 1994). The squared Pearson's r reflects the proportion of the variance explained. Cohen (1988) has used such effect sizes as a simple yardstick for the magnitude of effects, calling effect sizes small when less than 1% of the variance is explained, intermediate when 1%–9% of the variance is explained, and large when >25% of the variance is explained.

## 3 RESULTS

### 3.1 Infection experiment

**FIGURE 1:**
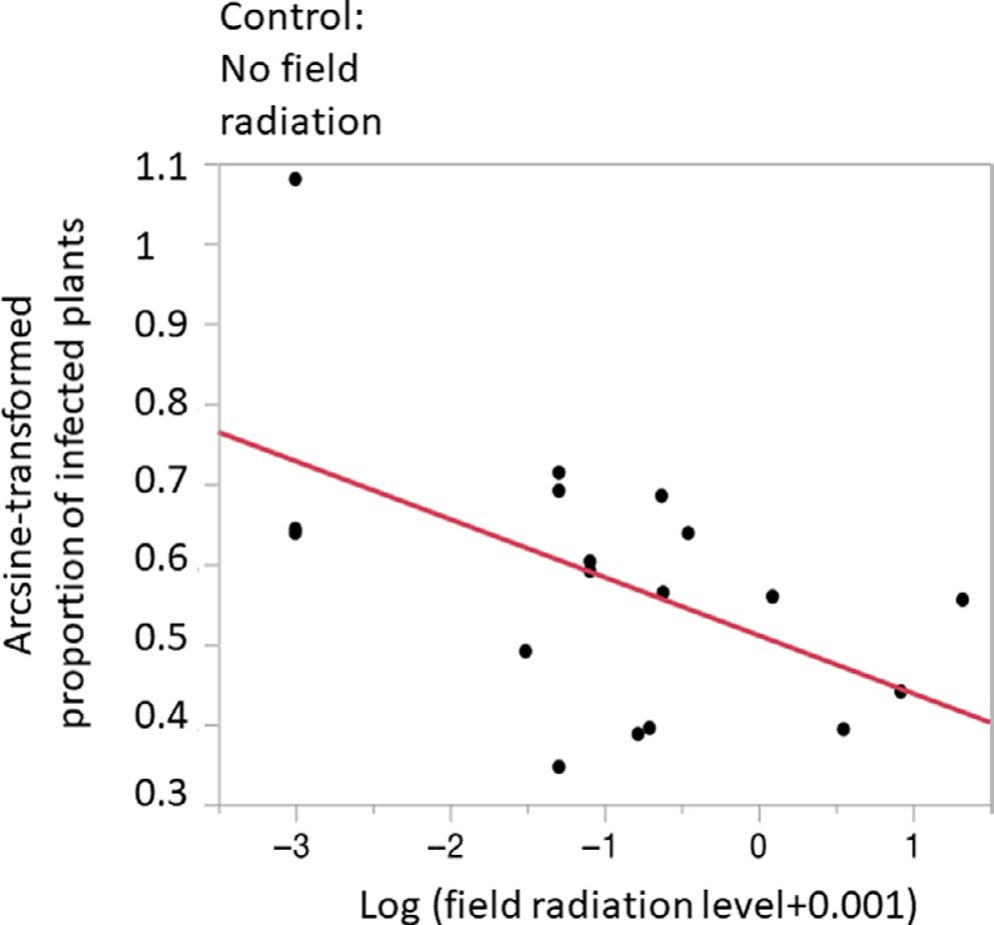
Proportion (arcsine‐transformed) of plants with disease symptoms following inoculation with *Microbotryum lychnidis‐dioicae* as a function of ambient radiation level at the collection site of the fungal strains (log‐transformed). Proportion infection is a single value of for a given strain, representing the total number of diseased plants over the total number of plants inoculated by this strain. Strains of the anther‐smut fungus from more contaminated sites infected plants less well than those from less contaminated or control, uncontaminated sites. *F*_(1,16)_ = 6.20, *p* = .024, slope ± *SE* = −0.069 ± 0.028, *R* ^2^ = .28, effect size = 0.528. The red line is the linear regression line. The control sample originating from sites without field radiation is indicated at left

None of the control plants became diseased. Of the inoculated plants, 534 flowered, 162 of which were diseased, yielding an infection rate of 30.3% of the plants that flowered. Only about half of both control and inoculated plants flowered, which is typical for *S. latifolia* in the first year of this perennial plant (Giraud, Jonot, & Shykoff, 2005; Kaltz, Gandon, Michalakis, & Shykoff, 1999), but at least 25 plants from each fungal strain treatment flowered, so estimates of infection rate are reliable. Infection success varied considerably, from 11.5% to 77.8%, across the 18 fungal strains (Table A2). Strains from more contaminated sites infected less well than those from pristine or less contaminated ones, as shown by the regression (Figure 1; *F*_(1,16)_ = 6.19, *p* = .024, slope ± *SE* = −0.069 ± 0.028, *R* ^2^ = .28) and a large effect size of 0.528 explaining 28% of the variance.

### 3.2 Irradiation experiment

Experimental radiation exposures of 2,000 Gy or more yielded almost no viable sporidial colonies. Therefore, we restricted our analyses to the controls and the 30‐ and 60‐min exposures (about 500 and 1,000 Gy total dose). Counts from the different plates from the same strain and irradiation treatment were significantly repeatable (control: *F*_(8,14)_ = 3.56, *p* = .018, large effect size = 0.45, accounting for 20% of the variance; 30‐min exposure: *F*_(8,15)_ = 10.76, *p* < .0001, large effect size = 0.65, accounting for 42% of the variance; 60‐min exposure: *F*_(8,18)_ = 27.68, *p* < .0001, large effect size = 0.78, accounting for 61% of the variance; Table A3). We therefore used means of repeated measures in further analyses.

**FIGURE 2:**
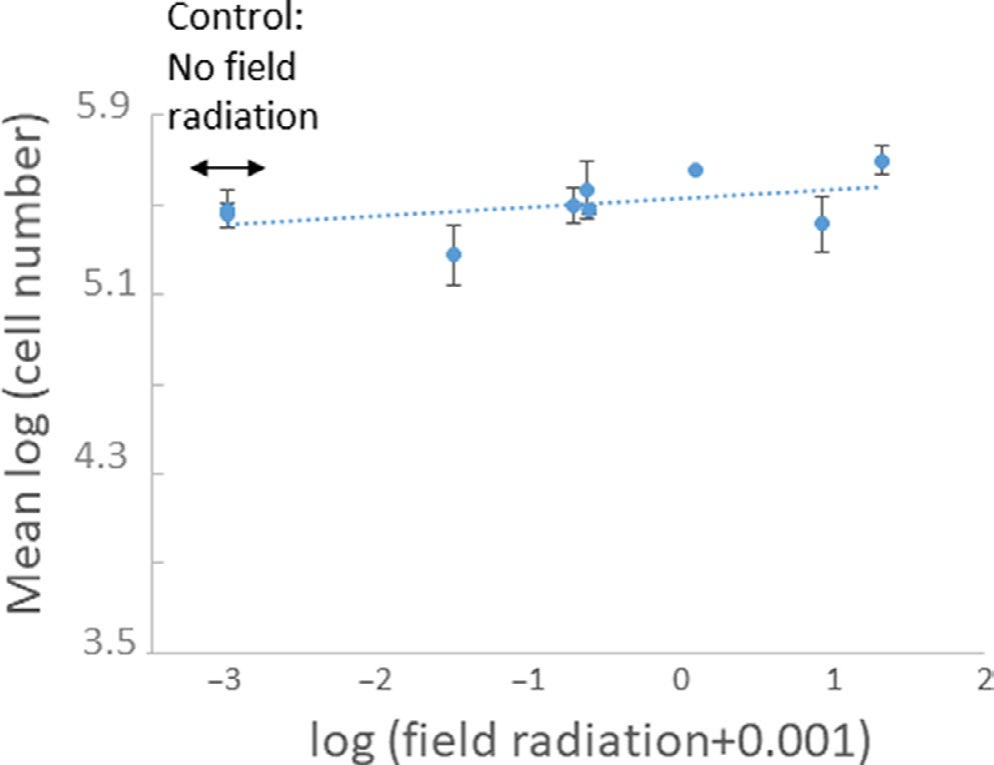
Number of viable sporidia of *Microbotryum lychnidis‐dioicae* strains in an aliquot of spore cultures from a single anther from the control treatment, that is, that had not been experimentally irradiated, plotted against the ambient radiation level at the collection site of the fungal strains. Bars represent standard deviations. The dotted line represents the linear regression, which is not significant (*F*_(1,7)_ = 1.98, *p* = .2, effect size = 0.47, *R* ^2^ = .22)

We found no significant relationship between the control cell count numbers and the level of contamination of the site from which the strain had been collected (*F*_(1,7)_ = 1.98, *R* ^2^ = .22, *p* = .2). However, the effect size was quite large, at 0.47, accounting for 22% of the variance, and the sporidia number actually increased, if anything, with field radiation level (Figure 2). Since we counted the same‐size aliquot for each sample after identical culture techniques of spores from a single anther, this suggests that those rare infections at sites with higher contamination levels produced diseased flowers with anthers bearing comparable or even higher numbers of spores with similar or higher viability compared with the more common infections in less contaminated sites.

**FIGURE 3:**
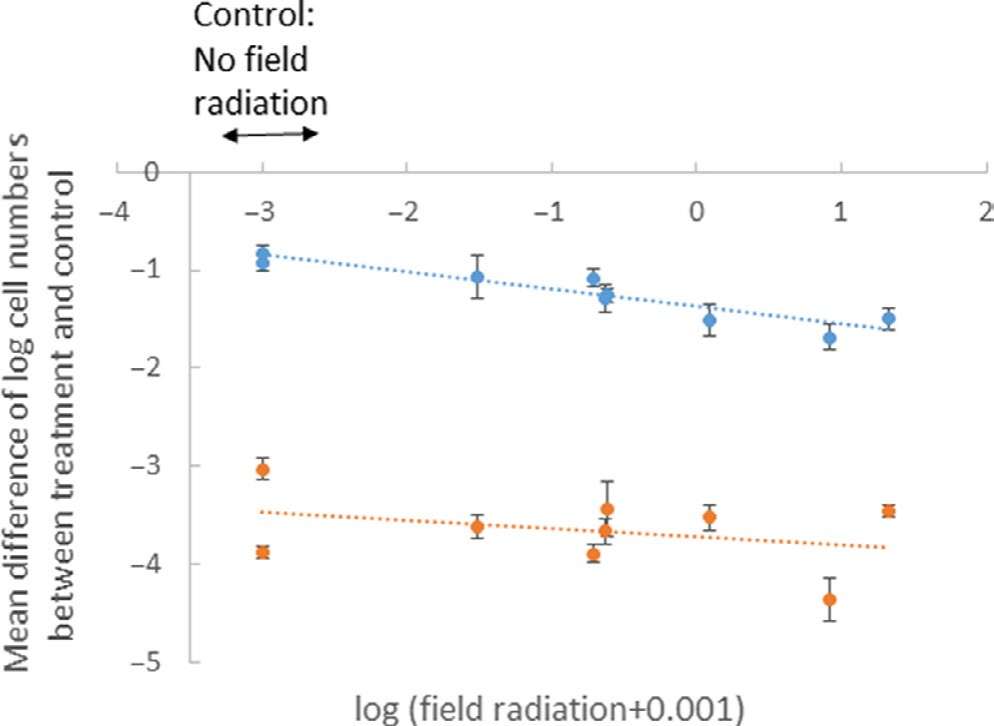
Effect of acute radiation exposure and degree of contamination at the field site from which *Microbotryum lychnidis‐dioicae* strains were collected on the viability of sporidia. Sporidial suspensions were exposed to a caesium‐137 γ source for 30 min (blue) or 60 min (red) at 17.178 Gy/min, resulting in total exposures of 515 or 1,031 Gy, respectively. Viable growing colonies were counted after being plated onto potato dextrose agar. Each point represents the difference in viability for each strain compared to its control that had not been exposed to radiation. Error bars represent standard deviations. Data were log‐transformed so a difference of −1 indicates a 10‐fold reduction in viability. The control sample originating from sites without field radiation is indicated at the left. ANCOVA revealed an effect of radiation treatment (*F*_(1,14)_ = 351.36, *p* < .0001) and of degree of radioactive contamination at the site of collection of the strains (*F*_(1,14)_ = 8.55, *p* = .01, slope ± *SE *= −0.13 ± 0.04, model *R* ^2^ = .96). The effect sizes for the corresponding effects were 0.98 for the treatment effect and 0.54 for degree of radioactive contamination at the site of strain collection. The dotted lines represent the linear regression for the mean difference in the log‐transformed viable cell number between each irradiated plate and each control plate of the same strain, against the ambient radiation level at the collection site of the fungal strains

Experimental radiation treatment affected sporidia viability, with fewer sporidial colonies growing from the samples following 30 or 60 min of acute exposure than following no radiation exposure, showing that all fungal strains suffered from being exposed to radiation (Figure 3). Sporidia showed lower viability following increased radiation exposure, with a large effect size of 0.98 accounting for 97% of the variance (main effect of treatment: *F*_(2,21)_ = 623.82, *p* < .0001; Figure 3), with a reduction in viability of over 10‐fold following exposure to 515 Gy and over 1000‐fold following 1,031 Gy exposure (Figure 3 with a log scale). Strains isolated from more contaminated sites showed significantly more, rather than less, negative effects of irradiation treatment (*F*_(1,21)_ = 8.55, *p* = .008) in a similar way for the two radiation doses; that is, there was no significant interaction between the factors (*F*_(2,21)_ = 2.90, *p* = .08; Figure 3). Effect size of contamination level was large at 0.54, accounting for 29% of the variance, and was intermediate for the interaction at 0.35, accounting for 12% of the variance.

## 4 DISCUSSION

The nuclear disaster at Chernobyl provides a case study for the effects of chronic ionizing radiation on fitness of living organisms and on their ability to tolerate or evolve resistance to such radiation. In a previous study, we found that neither the budding rate of haploid spores following meiosis nor the karyotype structure varied with increasing radiation levels at sampled sites (Aguileta et al., 2016). This is consistent with our findings in the present study, where we found no difference in sporidia counts as a function of the field radiation level in the treatment without experimental radiation. We further assessed here the ability of the same *M. lychnidis‐dioicae* strains to perform their whole life cycle up to the production of symptoms in the plants, that is, the production of anthers full of fungal spores. We found that fungal strains from more contaminated sites infected host plants less well, indicating lower fitness likely due to radiation exposure. Such lower infection ability helps to explain previous field prevalence data (Aguileta et al., 2016), in which *Silene latifolia* plants castrated by *M. lychnidis‐dioicae* were found to be rarer at more contaminated sites. The lower infection ability that we found experimentally for strains originating from sites with higher radiation levels indeed suggests that these strains are less able to cause disease in the field, which explains why the disease is rarer at more contaminated sites. The previously reported lower pollinator abundance at more contaminated sites (Aguileta et al., 2016) also likely contributes to lower spore transmission rates for the pollinator‐borne *M. lychnidis‐dioicae,* and therefore lower disease prevalence. The effect size we found for the influence of field contamination level on infection success (0.528, 28% variance explained) is by any measure large (Cohen, 1988; Møller & Jennions, 2002). These findings show the importance of investigating the entire life cycle to detect fitness effects and concur with previous studies in showing that the fitness of living organisms is affected by radiation after nuclear disasters (Møller & Mousseau, 2015). Our previous studies detected no significant increases in genome‐wide nonsynonymous substitution rates with increasing field radiation levels (Aguileta et al., 2016), but a few deleterious mutations can be sufficient for important fitness effects.

We further assessed here whether fungal individuals from more contaminated sites tolerated experimental radiation better or worse. Not surprisingly, none of the strains we tested were able to tolerate extremely high‐dose exposures of 2,,000 Gy or more, which is much higher than what they experience in the field sites where they grow. We found no evidence that resistance to radiation had evolved within contaminated populations. This apparent lack of adaptive evolution over a 28‐year time span is perhaps not surprising given that *Microbotryum* fungi have low effective population sizes (Gladieux et al., 2011) and no melanized structures. The survival rate of sporidia actually even decreased with increasing level of contamination at the site whence the strains came, which suggests that the chronic radiation exposure during their lifetime also negatively affected their resistance to further acute radiation exposure (Omar‐Nazir et al., 2018). One can indeed imagine that DNA damage accumulated in the field would render further damage more deleterious. It is still however possible that resistance to chronic exposure has evolved, but is not efficient against the high experimental doses that we applied in this study. Other structures of the fungus that are more long‐lived than the haploid sporidia (Schäfer et al., 2010), such as the dark teliospores, or the dikaryotic mycelium within the plants, could, indeed, be more resistant to radiation, but we have not yet tested this because the mycelium is not cultivable.

We calculated effect sizes to help understand the magnitude of the effects of radiation on infection success and haploid spore viability in the anther‐smut fungus *M. lychnidis‐dioicae*. The effect sizes estimated as Pearson's product–moment correlation coefficients were in the range 0.34–0.98 for the nine tests presented in Results. These effects accounted for 12% to 97% of the variance. Møller and Jennions (2002) reported a mean effect size of 5%–7% of the variance across all meta‐analyses in ecology and evolutionary biology. Hence, we can conclude that radiation, both experimental irradiation and the contamination level at the site from which these fungal strains were collected, had large, biologically meaningful effects on the biological processes we measured.

The findings of lower fitness of strains from more radiated sites and with no evidence of tolerance evolution to radiation generally concur with a previous meta‐analysis, showing that living organisms do not seem to rapidly evolve resistance to radiation exposure following nuclear disasters such as those in Chernobyl or Fukushima (Møller & Mousseau, 2016). Indeed, the microorganisms identified from highly radioactive sites in Chernobyl belong to groups characterized by resistance to UV damage as an adaptation to living on sunlight‐exposed surfaces, suggesting that their ability to tolerate radioactivity represents a preadaptation rather than an evolved response to the radioactivity in Chernobyl (Ragon, G, Moreira D, Møller AP, & López‐García P., 2011). Such adaptations may be related to efficient mechanisms for repairing damaged DNA, and for eliminating damaged proteins (Friedberg, Walker, & Siede, 1995). Fungi are generally known to have naturally high resistance to gamma radiation (Saleh et al., 1988), with basidiomycete and ascomycete yeasts identified as particularly resistant group (Shuryak, 2019; Zhdanova et al., 1991). Our results for *M. lychnidis‐dioicae* appear to concur with this. Indeed, this fungus seemed naturally quite resistant to radiation, with only a 10‐fold reduction in viability following 515 Gy exposure, compared with the fungus *Sporothrix schenckii*, for which no colonies could be recovered above exposure of 8 kGy (de Souza Lacerda, Martins, de Resende, & de Andrade, 2011). In general, fungi are extraordinarily resistant to ionizing radiation when compared to other eukaryotic organisms. Although there is considerable variation among species, cell type, and life history stage, the lethal dose needed to kill 50% of cells (i.e., LD50) ranges from 1 to over 3,000 Gy averaging around 1,500 Gy, while the LD100 (i.e., dose required to sterilize) can exceed 15,000 Gy in some species (Sommer, 1973). Studies on bacteria have found survival rates following 500 Gy radiation exposure that varied from about 10^–1^ to as low as 10^–6^ (Beblo‐Vranesevic et al., 2018). Bacteria associated with bird feathers from the Chernobyl area showed similar viability reduction at 500 Gy, but these bacteria were more resistant to higher doses of radiation than was *M. lychnidis‐dioicae* (Ruiz‐González et al., 2016). Overall, these results together with previous studies on microorganisms from Chernobyl show little evidence of adaptation to their radioactively contaminated environment (but see Ruiz‐González et al., 2016) despite the fact that microorganisms should show more rapid evolutionary responses than organisms with longer generation times and smaller effective population sizes. We clearly need additional studies of the long‐term evolutionary response to chronic environmental stresses such as the radioactive contamination caused by nuclear tests and accidents to understand and predict how organisms react to human‐induced environmental changes.

## CONFLICT OF INTEREST

None declared.

## AUTHOR CONTRIBUTIONS

**Sylvie Arnaise:** Data curation (equal); investigation (equal); methodology (equal); writing–original draft (supporting). **Jacqui Shykoff:** Data curation (equal); formal analysis (equal); investigation (equal); writing–original draft (equal); writing–review and editing (equal). **Anders Møller:** Formal analysis (equal); writing–review and editing (equal). **Timothy Mousseau:** Funding acquisition (equal); writing–review and editing (equal). **Tatiana Giraud:** Conceptualization (equal); supervision (equal); writing–original draft (equal).

## Data Availability

The data are available in Appendix Tables A1‐A3.

## APPENDIX 1

**TABLE A1:** Information on *Microbotryum lychnidis‐dioicae* strains used in this study: strain ID, GPS coordinates, date of collection, and radiation level measure (μSv/hr) at the collection site

Strain ID	Location	GPS coordinates	Date of collection	Radiation (μSv/hr)
1101‐1	Near Zalizia, Ukraine	N51°8′60″ 51.26523 E30°7′12″ 30.212083	September 2013	1.234
1101‐2	Near Zalizia, Ukraine	N51°8′60″ E30°7′12″	September 2013	0.240
1101‐3	Near Zalizia, Ukraine	N51°8′60″ E30°7′12″	September 2013	0.235
1102‐1	EcoCenter, Ukraine	N51°12′36″ 51.268583 E30°0′0″ 30.3253	September 2013	0.196
1102–2	EcoCenter, Ukraine	N51°12′36″ E30°0′0″	September 2013	0.165
1103‐3	Vesniane, Ukraine	N51°18′600″ 51.31 E 29°38′263″ 29,637716	September 2013	3.560
1105	Red Forest, Ukraine	N51°13′48″ 51.3873 E30°2′23.999″ 30.059716	September 2013	21.030
1106	Hilton, Ukraine	N51°32′378″ 51.53963333 E21°10′427″ 31.173783	September 2013	0.080
1109	Ecopolis, Ukraine	N51°23′355″ 51.38925 E30°04′225″ 30.070416	September 2013	0.050
1161	Chernobyl Village	N51°33′000″ 51.55 E31°11′212″ 31.18686667	June 2014	0.350
1162	Vesniane, Ukraine	N51°10′48″ 51.31 E30°22′12″ 29.637716	June 2014	8.350
1163	Ivankov, Ukraine	N51°13′48″ 50.94105 E30°1′11.999″ 28.9093	June 2014	0.080
1164	Voronkov, Ukraine	N50°7′48″ 50.21895 E30°31′48″ 30.89823	June 2014	0.030
1165‐1	Near Chernobyl, Ukraine	N51°12′36″ 51.53963 E30°0′0″ 31.173783	June 2014	0.050
1165‐2	Near Chernobyl, Ukraine	N51°12′36″ E30°0′0″	June 2014	0.050
1142‐2	Budaörs, Hungary	47°27′46.2″N 18°55′15.0″E 47.462819, 18.920830	June 2014	Control: 0
1191	Bingen‐am‐Rhein, Germany	49°57′43.9″N 7°54′11.5″E 49.962197, 7.903181	August 2014	Control: 0
1192	Monheim‐am‐Rhein, Germany	51°05′59.9″N 6°54′13.2"E 51.099971, 6.903672	August 2014	Control: 0

Note

In strain ID, the first number is the collection number of the population in the laboratory and the second number is the ID of the strain in the population, when applicable. All strains were collected before November 2014, so the Nagoya protocol does not apply.

**TABLE A2:** Diseased rates after artificial inoculation experiments by *Microbotryum lychnidis‐dioicae* strains on host seedlings of *Silene latifolia* grown from seeds collected on the Paris‐Saclay University campus (Orsay, France) in summer 2015, before French laws on Nagoya protocols were ratified: strain ID, radiation‐level measure (μSv/hr) at the collection site, groups of radiation levels, number of diseased plants (with fungal spores in anthers), total number of flowering plants, and percentage of diseased plants

Strain ID	Radiation (µSv/hr)	Groups of field radiation levels	No. of diseased plants	Total number of flowering plants	Percentage of diseased plant
1142‐2	0.00	Control	11	31	35.5
1191	0.00	Control	21	27	77.8
1192	0.00	Control	9	25	36.0
1164	0.030	Low	6	27	22.2
1109	0.050	Low	3	26	11.5
1165‐1	0.050	Low	13	32	40.6
1165‐2	0.050	Low	12	28	42.9
1163	0.080	Low	9	28	32.1
1106	0.080	Low	9	29	31.0
1102‐2	0.165	Low	4	28	14.3
1102‐1	0.196	Low	4	27	14.8
1101‐3	0.235	Low	10	25	40.0
1101‐2	0.240	Low	10	35	28.6
1161	0.350	Low	11	31	35.5
1101‐1	1.234	High	9	32	28.1
1103‐3	3.560	High	5	34	14.7
1162	8.350	High	6	33	18.2
1105	21.030	High	10	36	27. 8

**TABLE A3:** Results of experimental radiation on *Microbotryum lychnidis‐dioicae* strains: strain ID, radiation‐level measure (μSv/hr) at the collection site and its log transformation, and the mean difference in the log‐transformed viable cell number between each irradiated plate and each control plate of the same strain, for the 30‐min (515 kGy) and 60‐min (or 1,031 kGy) irradiation treatments, with standard deviations (*SD*) when count repeats were available

Strain ID	Radiation at origin	log (Radiation at origin + 0.001)	Mean log (number of viable cells) control ± *SD*	Mean (log number of viable cells in 30‐min exposure – log number of cells in control) ± *SD*	Mean (log number of viable cells in 60‐min exposure – log number of cells in control) ± *SD*
1142	0	−3.00	5.45 ± 0.08	−0.93 ± 0.08	−3.87 ± 0.06
1192	0	−3.00	5.48 ± 0.05	−0.82 ± 0.08	−3.03 ± 0.11
1164	0.03	−1.51	5.28 ± 0.13	−1.07 ± 0.22	−3.62 ± 0.12
1102‐1	0.20	−0.71	5.50 ± 0.08	−1.08 ± 0.08	−3.89 ± 0.09
1101‐3	0.24	−0.63	5.57 ± 0.13	−1.29 ± 0.13	−3.66 ± 0.12
1101‐2	0.24	−0.62	5.48 ± 0.02	−1.26 ± 0.06	−3.43 ± 0.27
1101‐1	1.23	0.09	5.65	−1.51 ± 0.16	−3.52 ± 0.13
1162	8.35	0.92	5.41 ± 0.12	−1.68 ± 0.12	−4.36 ± 0.22
1105	21.03	1.32	5.70 ± 0.07	−1.50 ± 0.11	−3.46 ± 0.06

## References

[ece36376-bib-0001] Aguileta, G. , Badouin, H. , Hood, M. E. , Møller, A. P. , Le Prieur, S. , Snirc, A. , … Giraud, T. (2016). Lower prevalence but similar fitness in a parasitic fungus at higher radiation levels near Chernobyl. Molecular Ecology, 25, 3370–3383. 10.1111/mec.13675 27136128

[ece36376-bib-0002] Akaike, H. (1981). Current contents. Engineering, Technology, and Applied Sciences, 12(51), 42.

[ece36376-bib-0003] Azizyan, A. A. , & Ter‐Hovhannesyan, A. R. (2010). Radiosensitivity of two strains of the codling moth *Cydia pomonella* (Linnaeus) (Lepidoptera: Tortricidae) originating from different elevations in Armenia. Journal of Applied Entomology, 134, 227–233.

[ece36376-bib-0004] Beblo‐Vranesevic, K. , Bohmeier, M. , Perras, A. K. , Schwendner, P. , Rabbow, E. , Moissl‐Eichinger, C. , … Rettberg, P. (2018). Lack of correlation of desiccation and radiation tolerance in microorganisms from diverse extreme environments tested under anoxic conditions. FEMS Microbiology Letters, 365(6). 10.1093/femsle/fny044 PMC5939664 29474542

[ece36376-bib-0005] Boubriak, I. I. , Grodzinsky, D. M. , Polischuk, V. P. , Naumenko, V. D. , Gushcha, N. P. , Micheev, A. N. , … Osborne, D. J. (2008). Adaptation and impairment of DNA repair function in pollen of *Betula verrucosa* and seeds of *Oenothera biennis* from differently radionuclide‐contaminated sites of Chernobyl. Annals of Botany, 101, 267–276. 10.1093/aob/mcm276 17981881 PMC2711018

[ece36376-bib-0006] Burnham, K. P. , & Anderson, D. R. (2002). Model selection and multi‐model inference. New York, NY: Springer.

[ece36376-bib-0007] Byrne, R. T. , Klingele, A. J. , Cabot, E. L. , Schackwitz, W. S. , Martin, J. A. , Martin, J. , … Cox, M. M. (2014). Evolution of extreme resistance to ionizing radiation via genetic adaptation of DNA repair. eLife, 3, e01322 10.7554/eLife.01322 24596148 PMC3939492

[ece36376-bib-0008] Cohen, J. (1988). Statistical power analysis for the behavioral sciences. Hillsdale, MI: Erlbaum.

[ece36376-bib-0009] Cordeiro, A. R. , Marques, E. K. , & Veiga‐Neto, A. J. (1973). Radioresistance of a natural population of *Drosophila willistoni* living in a radioactive environment. Mutation Research/Fundamental and Molecular Mechanisms of Mutagenesis, 19, 325–329. 10.1016/0027-5107(73)90233-9 4796403

[ece36376-bib-0010] de Souza Lacerda, C. M. , Martins, E. M. D. N. , de Resende, M. A. , & de Andrade, A. S. R. (2011). Gamma radiation effects on *Sporothrix schenckii* yeast cells. Mycopathologia, 171, 395–401. 10.1007/s11046-011-9395-9 21327789

[ece36376-bib-0011] Einor, D. , Bonisoli‐Alquati, A. , Costantini, D. , Mousseau, T. A. , & Møller, A. P. (2016). Ionizing radiation, antioxidant response and oxidative damage: A meta‐analysis. Science of the Total Environment, 548–549, 463–471. 10.1016/j.scitotenv.2016.01.027 26851726

[ece36376-bib-0012] Ellegren, H. , Lindgren, G. , Primmer, C. R. , & Møller, A. P. (1997). Fitness loss and germline mutations in barn swallows breeding in Chernobyl. Nature, 389, 593–596. 10.1038/39303 9335497

[ece36376-bib-0013] Everitt, B. S. (2002). The Cambridge dictionary of statistics. Cambridge, UK: Cambridge University Press.

[ece36376-bib-0014] Friedberg, E. C. , Walker, G. C. , & Siede, W. (1995). DNA repair and mutagenesis. Washington, DC: ASM Press.

[ece36376-bib-0015] Galván, I. , Bonisoli‐Alquati, A. , Jenkinson, S. , Ghanem, G. , Wakamatsu, K. , Mousseau, T. A. , & Møller, A. P. (2014). Chronic exposure to low‐dose radiation at Chernobyl favours adaptation to oxidative stress in birds. Functional Ecology, 28, 1387–1403. 10.1111/1365-2435.12283

[ece36376-bib-0016] Garnier‐Laplace, J. , Geras’kin, S. , Della‐Vedova, C. , Beaugelin‐Seiller, K. , Hinton, T. G. , Real, A. , & Oudalova, A. (2013). Are radiosensitivity data derived from natural field conditions consistent with data from controlled exposures? A case study of Chernobyl wildlife chronically exposed to low dose rates. Journal of Environmental Radioactivity, 121, 12–21. 10.1016/j.jenvrad.2012.01.013 22336569

[ece36376-bib-0017] Geras’kin, S. A. , Kim, J. K. , Oudalova, A. A. , Vasiliyev, D. V. , Dikareva, N. S. , Zimin, V. L. , & Dikarev, V. G. (2005). Bio‐monitoring the genotoxicity of populations of Scots pine in the vicinity of a radioactive waste storage facility. Mutation Research/Genetic Toxicology and Environmental Mutagenesis, 583, 55–66. 10.1016/j.mrgentox.2005.02.003 15866466

[ece36376-bib-0018] Geras’kin, S. , Oudalova, A. , Dikareva, N. , Spiridonov, S. , Hinton, T. , Chernonog, E. , & Garnier‐Laplace, J. (2011). Effects of radioactive contamination on Scots pines in the remote period after the Chernobyl accident. Ecotoxicology, 20, 1195–1208. 10.1007/s10646-011-0664-7 21451948

[ece36376-bib-0019] Geras'kin, S. , Evseeva, T. , & Oudalova, A. (2013). Effects of long‐term chronic exposure to radionuclides in plant populations. Journal of Environmental Radioactivity, 121, 22–32. 10.1016/j.jenvrad.2012.03.007 22483340

[ece36376-bib-0020] Giraud, T. , Jonot, O. , & Shykoff, J. A. (2005). Selfing propensity under choice conditions in a parasitic fungus, *Microbotryum violaceum*, and parameters influencing infection success in artificial inoculations. International Journal of Plant Sciences, 166, 649–657.

[ece36376-bib-0021] Gladieux, P. , Vercken, E. , Fontaine, M. C. , Hood, M. E. , Jonot, O. , Couloux, A. , & Giraud, T. (2011). Maintenance of fungal pathogen species that are specialized to different hosts: Allopatric divergence and introgression through secondary contact. Molecular Biology and Evolution, 28(1), 459–471. 10.1093/molbev/msq235 20837605

[ece36376-bib-0022] Gladyshev, E. , & Meselson, M. (2008). Extreme resistance of bdelloid rotifers to ionizing radiation. Proceedings of the National Academy of Sciences of the United States of America, 105, 5139–5144. 10.1073/pnas.0800966105 18362355 PMC2278216

[ece36376-bib-0023] Holliday, R. (2004). Early studies on recombination and DNA repair in *Ustilago maydis* . DNA Repair, 3, 671–682. 10.1016/j.dnarep.2004.02.002 15135734

[ece36376-bib-0024] Kaltz, O. , Gandon, S. , Michalakis, Y. , & Shykoff, J. A. (1999). Local maladaptation in the anther‐smut fungus *Microbotryum violaceum* to its host plant *Silene latifolia*: Evidence from a cross‐inoculation experiment. Evolution, 53, 395–407. 28565431 10.1111/j.1558-5646.1999.tb03775.x

[ece36376-bib-0025] Kaltz, O. , & Shykoff, J. A. (2002). Within‐ and among‐population variation in infectivity, latency and spore production in a host–pathogen system. Journal of Evolutionary Biology, 15, 850–860.

[ece36376-bib-0026] Kovalchuk, I. , Abramov, V. , Pogribny, I. , & Kovalchuk, O. (2004). Molecular aspects of plant adaptation to life in the Chernobyl zone. Plant Physiology, 135, 357–363. 10.1104/pp.104.040477 15133154 PMC429389

[ece36376-bib-0027] Kovalchuk, O. , Burke, P. , Arkhipov, A. , Kuchma, N. , James, S. J. , Kovalchuk, I. , & Pogribny, I. (2003). Genome hypermethylation in *Pinus sylvestris* of Chernobyl: A mechanism for radiation adaptation? Mutation Research, 529, 13–20. 12943916 10.1016/s0027-5107(03)00103-9

[ece36376-bib-0028] Kovalchuk, O. , Dubrova, Y. E. , Arkhipov, A. , Hohn, B. , & Kovalchuk, I. (2000). Germline DNA: Wheat mutation rate after Chernobyl. Nature, 407, 583–584. 10.1038/35036692 11034198

[ece36376-bib-0029] Lehmann, P. , Boratynski, Z. , Mappes, T. , Mousseau, T. A. , & Møller, A. P. (2016). Cataract frequency, fecundity and background radiation in natural mammalian populations from Chernobyl. Scientific Reports, 6, 19974. 26814168 10.1038/srep19974 PMC4728484

[ece36376-bib-0030] López‐Villavicencio, M. , Jonot, O. , Coantic, A. , Hood, M. E. , Enjalbert, J. , & Giraud, T. (2007). Multiple infections by the anther smut pathogen are frequent and involve related strains. PLoS Path, 3(11), e176 10.1371/journal.ppat.0030176 PMC2077905 18020704

[ece36376-bib-0031] Mironenko, N. V. , Alekhina, I. A. , Zhdanova, N. , & Bula, S. A. (2000). Intraspecific variation in gamma‐radiation resistance and genomic structure in the filamentous fungus *Alternaria alternata*: A case study of strains Inhabiting Chernobyl reactor No. 4. Ecotoxicology and Environmental Safety, 45, 177–187. 10.1006/eesa.1999.1848 10648134

[ece36376-bib-0032] Møller, A. P. , Bonisoli‐Alquati, A. , & Mousseau, T. A. (2013). High frequency of albinism and tumours in free‐living birds around Chernobyl. Mutation Research, 757, 52–59. 23850808 10.1016/j.mrgentox.2013.04.019

[ece36376-bib-0033] Møller, A. P. , Bonisoli‐Alquati, A. , Mousseau, T. A. , & Rudolfsen, G. (2012). Aspermy, sperm quality and radiation in Chernobyl birds. PLoS ONE, 9, e100296 10.1371/journal.pone.0100296 PMC4070951 24963711

[ece36376-bib-0034] Møller, A. P. , Bonisoli‐Alquati, A. , Rudolfsen, G. , & Mousseau, T. A. (2012). Elevated mortality among birds in Chernobyl as judged from skewed age and sex ratios. PLoS ONE, 7, e35223 10.1371/journal.pone.0035223 22514722 PMC3324427

[ece36376-bib-0035] Møller, A. P. , & Jennions, M. D. (2002). How much variance can be explained by ecologists and evolutionary biologists? Oecologia, 132, 492–500. 10.1007/s00442-002-0952-2 28547634

[ece36376-bib-0036] Møller, A. P. , & Mousseau, T. A. (2011). Conservation consequences of Chernobyl and other nuclear accidents. Biological Conservation, 144, 2787–2798. 10.1016/j.biocon.2011.08.009

[ece36376-bib-0037] Møller, A. P. , & Mousseau, T. A. (2015). Strong effects of ionizing radiation from Chernobyl on mutation rates. Scientific Reports, 5, 8363 10.1038/srep08363 25666381 PMC4322348

[ece36376-bib-0038] Møller, A. P. , & Mousseau, T. A. (2016). Are organisms adapting to ionizing radiation at Chernobyl? Trends in Ecology & Evolution, 31, 281–289. 10.1016/j.tree.2016.01.005 26868287

[ece36376-bib-0039] Mousseau, T. A. , Milinevsky, G. , Kenney‐Hun, J. , & Møller, A. P. (2014). Highly reduced mass loss rates and increased litter layer in radioactively contaminated areas. Oecologia, 175, 429–437. 10.1007/s00442-014-2908-8 24590204

[ece36376-bib-0040] Neyman, J. (1937). Outline of a theory of statistical estimation based on the classical theory of probability. Philosophical Transactions of the Royal Society of London. Series A, 236, 333–380.

[ece36376-bib-0041] Omar‐Nazir, L. , Shi, X. , Møller, A. P. , Mousseau, T. , Byun, S. , Hancock, S. , … Mothersill, C. (2018). Long‐term effects of ionizing radiation after the Chernobyl accident: Possible contribution of historic dose. Environmental Research, 165, 55–62. 10.1016/j.envres.2018.04.005 29665465

[ece36376-bib-0042] Pimentel, A. E. , Levine, L. , Cruces, M. P. , & Salceda, V. M. (2003). Radioresistance of sibling *Drosophila* species from Laguna Verde, Veracruz, México. International Journal of Radiation Biology, 79, 1003–1009. 14713578 10.1080/09553000310001632967

[ece36376-bib-0043] Ragon, M. R. , Restoux, G. , Moreira, D. , Møller, A. P. , & López‐García, P. (2011). Sunlight‐exposed biofilm microbial communities are naturally resistant to Chernobyl ionizing‐radiation levels. PLoS ONE, 6, e21764. 21765911 10.1371/journal.pone.0021764 PMC3135598

[ece36376-bib-0044] Rivasseau, C. , Farhi, E. , Atteia, A. , Couté, A. , Gromova, M. , & de Gouvion Saint Cyr, D. , … Bligny, R. (2013). An extremely radioresistant green eukaryote for radionuclide bio‐decontamination in the nuclear industry. Energy & Environmental Science, Royal Society of Chemistry, 6, 1230–1239.

[ece36376-bib-0045] Rosenthal, R. (1994). Parametric measures of effect size In Cooper H., & Hedges L. V. (Eds.), The Handbook of research synthesis (pp. 231–244). New York, NY: Russell Sage Foundation New York.

[ece36376-bib-0046] Ruiz‐González, M. , Czirják, G. , Genevaux, P. , Møller, A. P. , Mousseau, T. A. , & Heeb, P. (2016). Resistance of feather‐associated bacteria to intermediate levels of ionizing radiation near Chernobyl. Scientific Reports, 6, 22969. 26976674 10.1038/srep22969 PMC4792135

[ece36376-bib-0047] Saleh, Y. G. , Mayo, M. S. , & Ahearn, D. G. (1988). Resistance of some common fungi to gamma irradiation. Applied and Environmental Microbiology, 54, 2134–2135. 3178216 10.1128/aem.54.8.2134-2135.1988 PMC202816

[ece36376-bib-0048] Schäfer, A. M. , Kemler, M. , Bauer, R. , & Begerow, D. (2010). The illustrated life cycle of *Microbotryum* on the host plant *Silene latifolia* . Botany‐Botanique, 88, 875–885.

[ece36376-bib-0049] Shuryak, I. (2019). Review of microbial resistance to chronic ionizing radiation exposure under environmental conditions. Journal of Environmental Radioactivity, 196, 50–63. 10.1016/j.jenvrad.2018.10.012 30388428

[ece36376-bib-0050] Sokal, R. R. , & Rohlf, F. J. (1995). Biometry: The principles and practice of statistics in biological research (3rd ed.). New York, NY: W.H. Freeman and Co..

[ece36376-bib-0051] Sommer, N. (1973). The effect of ionising radiation on fungi In Manual on Radiation Sterilisation of Medical and Biological Materials, IAEA Technical Reports Series No. 149, (pp. 73–80).

[ece36376-bib-0052] Syomov, A. B. , Ptitsyna, S. N. , & Sergeeva, S. A. (1992). Analysis of DNA strand break induction and repair in plants from the vicinity of Chernobyl. Science of the Total Environment, 12, 1–8. 10.1016/0048-9697(92)90232-H 1574698

[ece36376-bib-0053] Vaisnav, M. , Xing, C. , Ku, H.‐C. , Hwang, D. , Stojadinovic, S. , Pertsemlidis, A. , & Abrams, J. M. (2014). Genome‐wide association analysis of radiation resistance in *Drosophila melanogaster* . PLoS ONE, 9, e104858. 25121966 10.1371/journal.pone.0104858 PMC4133248

[ece36376-bib-0054] Vercken, E. , Fontaine, M. , Gladieux, P. , Hood, M. E. , Jonot, O. , & Giraud, T. (2010). Glacial refugia in pathogens: European genetic structure of anther smut pathogens on *Silene latifolia* and *S. dioica* . PLoS Path, 6, e1001229. 10.1371/journal.ppat.1001229 PMC3002987 21187901

[ece36376-bib-0055] Zhdanova, N. N. , Vasilevskaia, A. I. , Artyshkova, L. V. , Gavriliuk, V. I. , Lashko, T. N. , & Sadovnikov, S. (1991). Complexes of soil micromycetes in the area of the influence of the Chernobyl atomic electric power station. Mikrobiol Zh, 53, 3–9. 1753885

[ece36376-bib-0056] Zhdanova, N. N. , Zakharchenko, V. A. , Vember, V. V. , & Nakonechnaya, L. T. (2000). Fungi from Chernobyl: Mycobiota of the inner regions of the containment structures of the damaged nuclear reactor. Mycological Research, 104, 1421–1426. 10.1017/S0953756200002756

